# Machine-learning ready data on the thermal power consumption of the Mars Express Spacecraft

**DOI:** 10.1038/s41597-022-01336-z

**Published:** 2022-05-24

**Authors:** Matej Petković, Luke Lucas, Jurica Levatić, Martin Breskvar, Tomaž Stepišnik, Ana Kostovska, Panče Panov, Aljaž Osojnik, Redouane Boumghar, José A. Martínez-Heras, James Godfrey, Alessandro Donati, Sašo Džeroski, Nikola Simidjievski, Bernard Ženko, Dragi Kocev

**Affiliations:** 1 Bias Variance Labs, Ljubljana, Slovenia; 2 Jožef Stefan Institute, Ljubljana, Slovenia; 3 LSE Space GmbH, Gilching, Germany; 4 European Space Agency – ESA, ESOC, Darmstadt, Germany; 5 Solenix GmbH, Darmstadt, Germany; 6 University of Cambridge, Cambridge, UK

**Keywords:** Computational science, Aerospace engineering

## Abstract

We present six datasets containing telemetry data of the Mars Express Spacecraft (MEX), a spacecraft orbiting Mars operated by the European Space Agency. The data consisting of context data and thermal power consumption measurements, capture the status of the spacecraft over three Martian years, sampled at six different time resolutions that range from 1 min to 60 min. From a data analysis point-of-view, these data are challenging even for the more sophisticated state-of-the-art artificial intelligence methods. In particular, given the heterogeneity, complexity, and magnitude of the data, they can be employed in a variety of scenarios and analyzed through the prism of different machine learning tasks, such as multi-target regression, learning from data streams, anomaly detection, clustering, etc. Analyzing MEX’s telemetry data is critical for aiding very important decisions regarding the spacecraft’s status and operation, extracting novel knowledge, and monitoring the spacecraft’s health, but the data can also be used to benchmark artificial intelligence methods designed for a variety of tasks.

## Abstract (summary)

**Table:** 

Measurement(s)	electric current
Technology Type(s)	current readings in spacecraft housekeeping telemetry
Sample Characteristic - Environment	outer space

## Background & Summary

The Mars Express (MEX) spacecraft has been orbiting and exploring Mars since 2003. Operated by the European Space Agency (ESA), from its European Space Operations Centre in Darmstadt, Germany – it continues to be a critical asset for a plethora of scientific discoveries. These include historical traces of water across the planet (i.e., a groundwater system^1^); showing that Mars once possessed an environment that might have been suitable for life; the presence of minerals that can form only in the presence of water^2^; detection of underground water-ice deposits^3,4^; the most complete map of the chemical composition of the Mars atmosphere (indications of the presence of methane - a gas related to active volcanism and biochemical processes)^5–7^; first global map of Martian ionosphere^8^; study on the plasma acceleration above Martian magnetic anomalies and the effects of solar-wind (i.e., study on Martian magnetosphere and exosphere)^9,10^; a wealth of three-dimensional renders of the surface^11^; and a study of the innermost moon Phobos in unprecedented detail^12^. Last, but not least, MEX provides relay communication services between Earth and the NASA assets on the Mars surface.

MEX hosts several scientific instruments (https://www.esa.int/Science_Exploration/Space_Science/Mars_Express/Mars_Express_instruments) that are used to perform: 1) imaging studies of the surface and subsurface of Mars, 2) atmosphere, 3) ionosphere and 4) plasma studies, 5) studies of gravity on Mars and 6) the solar corona, and finally 7) relay of data communication to Earth via a radio link. These instruments, together with the remaining on-board equipment, need to be kept in their operating temperature ranges (from –180°*C* for some instruments to as much as room temperature for other instruments). The autonomous on-board thermal system, containing 33 electrical heaters, controls the temperature of different parts of the spacecraft, and therefore is crucial to ensure safe and healthy exploitation of the spacecraft’s potential for scientific operations.

MEX is powered by electricity generated by its solar arrays and stored in batteries for use during the eclipse periods. The autonomous thermal system of MEX, through the 33 individual thermal power lines supplying the heaters, consumes a significant amount of the total available electric power, thus leaving only a small portion available for science operations. The better the thermal system optimizes its consumption, the more power remains for science. Given the age of the spacecraft, monitoring its condition, health, and status strongly influences the longevity of the MEX mission^13–17^. The activity of different heaters depends both on the instruments that are used at a given moment as well as the outer conditions of the spacecraft, e.g., the spacecraft is exposed to the Sun or being in the shadow of Mars. Since the thermal subsystem is autonomous, the potential power consumption, under given conditions, needs to be estimated in advance. By doing so, one can further estimate the amount of residual power left for the scientific operations of the MEX mission.

The data presented here document the activity of the thermal subsystem through the prism of the power consummation of the individual 33 thermal units. It covers the period from 22. 8. 2008 to 14. 4. 2014, i.e., three full Martian years (2062 Earth days). It describes the state of MEX through time at different time resolutions $\Delta t\in \{1,5,10,15,30,60\}$ (minutes). In particular, we present *Machine Learning (ML) ready* datasets associated with each of the time resolutions individually. Each datum (row) in a given dataset provides the values of different descriptive variables (features) for a given time interval $[t,t+\Delta t)$. The variables belong to five groups that measure and document different aspects of the spacecraft’s activity in this period:

- *Energy Influx*: Each feature in this group accounts for the amount of solar energy incident upon each of the seven surfaces of MEX (solar panels and the six sides of the central cube). They also consider the orientation of the spacecraft, i.e., the angle of the exposure to the Sun of a given spacecraft surface, the power of the Sun at MEX’s position, and possible celestial bodies that could cast a shadow on MEX (Mars, Phobos, and Deimos).
- *Flight time-line* (FTL): These features identify the potential pointing events (e.g., towards Mars, Earth, etc.) happening at a given time. Since communication with Earth consumes a considerable amount of energy, one of the features also describes the state of the radio transmitter (turned on or off).
- *Detailed mission operation plan* (DMOP): These features specify the time since issuing a given command to one of the MEX’s subsystems and the time since the last activity of that subsystem.
- *Additional positional data*: These features carry specific information about the astronomical data for a given position, e.g., the distance between Mars and Earth, the value of the solar constant, etc.
- *Power lines*: Each feature provides the values for the amount of electrical current running through a given power line at a given time point.

The presented data are crucial for analyzing MEX’s behavior, ensuring better exploitation of the on-board equipment, and keeping the spacecraft and the equipment safe and healthy. However, the benefits from the data extend beyond the spacecraft-operations community. In particular, these data is typically used for a variety of analysis tasks that include mission planning (i.e., navigating the spacecraft), trajectory and orbit planning; scheduling scientific experiments; as well as monitoring the health of subsystems and the spacecraft as a whole. Given the amount of data and the complexity of the tasks, coupled with the importance of extending MEX’s mission - this allows for tackling problems from different aspects, spanning from various areas of AI such as optimization, decision support, planning, and machine learning.

## Methods

We start by describing the feature engineering process that takes us from the raw data to the ML-ready (or more generally, AI-ready) data. The raw spacecraft data come in several parts. The telemetry data, that comprise the descriptive features, consist of:

1. *Solar aspect angles* (SAA) data contain the angles between the line *Sun–MEX* and the axes of the local coordinate system of MEX, and the angle between the line *Sun–MEX* and the normal vector of solar panels, see Fig. 1(a). These data are used for calculating the *Energy Influx Features*;

**Fig. 1:**
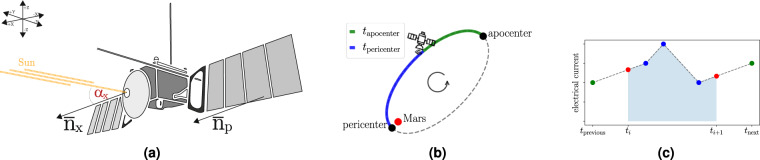
Illustrations of how we calculate the descriptive features. **(a)** The solar aspect angles give the orientation of the spacecraft. The angle between the line Sun-MEX and the normal vector to the front side of the cube ($${\alpha }_{x}$$), is shown. **(b)** A conceptual illustration of the elliptical orbit of MEX with Mars as a focal point. The two features $${t}_{{\rm{pericenter}}}$$ and $${t}_{{\rm{apocenter}}}$$ give the approximate position of MEX in the orbit. In this example, they give the (normalized) time since the last passing through the pericenter and the (normalized) time until the next passing through the apocenter. The sum of the values of the two features is always 1.0. Note that the illustration is not to scale. **(c)** An illustration of the preprocessing of the electrical currents. The known measurements on the interval $$[{t}_{i},{t}_{i+1})$$ (blue dots) and the first measurement before and after this interval (green dots) define the linearly interpolated curve from which the values at the different boundaries (red dots) are taken. The area under that curve (blue-shaded area), divided by the length of the interval Δ*t*, is the average value of the electrical current for the given time interval.
2. *Long-term* (LT) data give the values of physical quantities that can be computed far into the future, e.g., the distance between Mars and Earth, and the value of solar constant at Mars;
3. *Flight dynamics timeline events* data, containing the pointing and action commands that change the altitude or the orbit of the spacecraft. More specifically, they contain logs of pointing events and their time ranges, where simultaneous events are also possible. These can affect the thermal status of MEX due to the use of heat-generating equipment and changes in solar illumination.
4. *Detailed mission operation plans* (DMOP) document the time at which different commands have been issued, together with the subsystem to which the command is issued. Since some on-board instruments and software are proprietary, belonging to different parties, particular details regarding the specific commands and instruments have been anonymized. However, general descriptions of the command groups are provided with the data.
5. *Event* (EVT) data list the events related to the orbit of MEX, such as entering/exiting the shadow of Mars and passing through the extreme points (apo- and pericenter) of the orbit.

The remaining part contains the power consumption measurements. It provides the measured values of electrical current through each of the 33 power lines, from which the target variables (features) are derived. The names of these variables contain the fixed prefix “NPWD”, followed by a four-digit number for each the power line. Details about the location of each of the 33 power lines, relative to the spacecraft, are provided in the supplementary material. Given a time-resolution Δ*t* (of length 1, 5, 10, 15, 30 and 60 minutes), we derive values for every descriptive and target feature, in the respective time interval $$[{t}_{i},{t}_{i+1})$$ for the respective length. In the remainder, we provide further details on the procedures used to compute these values for each feature group.

### Energy Influx Features

Given the solar constant $$c(t)\;[W\,/\,{m}^{2}]$$, the area $${A}_{s}\;[{m}^{2}]$$ of a surface *s* (e.g., solar panels) exposed to the Sun, and the angle $$\alpha (t)$$ between the normal vector of that surface and the Sun direction, the amount of energy $${E}_{i,s}$$, collected by the surface in the time interval $$[{t}_{i},{t}_{i+1})$$, is computed in three steps. First, the adjusted area of the surface, i.e., its area in the direction of the Sun, is computed as $${\widehat{A}}_{s}(t)={A}_{s}{\rm{\max }}\{0,{\rm{\cos }}\,{\alpha }_{s}(t)\}$$. Next, the umbra coefficient $$U(t)[1]$$ is introduced (with the value 1, if MEX is not in shadow, 1/2 if it is in penumbra (half-shadow), and 0 if it is in umbra (shadow)) and the adjusted solar constant $$\widehat{c}(t)=U(t)c(t)$$ is computed. Finally, the energy $${E}_{s}(i)$$ can be computed as

1 $${E}_{s}(i)={\int }_{{t}_{i}}^{{t}_{i+1}}{\widehat{A}}_{s}(t)\widehat{c}(t){\rm{d}}t.$$

This is done for all six sides of the MEX cube and the solar panels. For a given surface, the values *α*(*t*) are taken from the SAA data. The value *c*(*t*) is taken from LT data, whereas the values of the umbra coefficient *U*(*t*) are determined from the EVT data. We linearly interpolate the values ${\widehat{A}}_{s}(t)$, since the values of *α*(*t*) are not known for all times *t*, but are logged by MEX once or twice a minute. When computing the integral from (1), we assume that ${A}_{s}=1\,{m}^{2}$, since in this machine-learning context the actual scale of the variables is not important, but rather their relationship. Solving the integral results in *E*_*s*_ with values expressed as $joules\;per\;sq.\;meter\;\left(J\,/{m}^{2}\right)$. Note that reflections, such as spacecraft-spacecraft and planet-spacecraft, and other thermal emissions of these bodies are neglected in the computation.

Since the activity of the heaters, at a given moment, also depends on the energy influx in the past, we also define historic energy influx features

2 $${H}_{s,n,w}(i)=\mathop{\sum }\limits_{j=1}^{n}\,{w}^{j}{E}_{s}\left(i-j\right)$$

for different values of a window size parameter *n*>0 and a decay parameter $w\in (0,1]$. The parameter *n* controls the relevance of past data, whereas the decay parameter *w* controls how quickly the influence of the historic data decreases. In the 1-minute resolution dataset, we use $n\in {{\mathscr{N}}}_{1}=\{4,16,32,64,128\}$ minutes of historic data, i.e., between 4 minutes and 128 minutes (approximately two hours). For the other dataset resolutions Δ*t*, we map the values from ${{\mathscr{N}}}_{1}$ to their closest positive multipliers of Δ*t* and use the corresponding values of *n*, i.e., ${{\mathscr{N}}}_{\Delta t}=\{{\rm{\max }}(1,round({n}_{1}/\Delta t))| {n}_{1}\in {{\mathscr{N}}}_{1}\}$. For example, the 10-minute resolution dataset uses $n\in \{1,2,3,6,13\}$. The values of the parameter *w* were the same for all time resolutions and were set to $w\in \{1.0,0.9,0.75,0.5,0.25\}$. The values ${H}_{s,n,w}(i)$ are non-normalized versions of exponential moving averages. Normalization of the values is not necessary here, since this only changes the scale of the features. These parameters and values were selected based on the domain knowledge provided by the spacecraft operators involved in the study.

### FTL Features

FTL data comprise the pointing events, together with information of whether the radio was used or not. Each pointing event *e* is described as a triplet $$e=({t}_{{\rm{start}}},{t}_{{\rm{end}}},p)$$, where $$[{t}_{{\rm{start}}},{t}_{{\rm{end}}})$$ is the time span of the event, and *p* is the point of interest, e.g., Earth or Mars. For every point *p*, we construct a feature. Its value within the time interval $$[{t}_{i},{t}_{i+1})$$ is calculated as the proportion of the time within this interval during which the pointing happend, i.e.,

3 $${F}_{p}(i)=\sum _{\left({t}_{{\rm{start}}},{t}_{{\rm{end}}},p\right)}\frac{\left|\left[{t}_{{\rm{start}}},{t}_{{\rm{end}}}\right)\cap \left[{t}_{i},{t}_{i+1}\right)\right|}{\left|\left[{t}_{i},{t}_{i+1}\right)\right|}=\frac{1}{\Delta t}\sum _{\left({t}_{{\rm{start}}},{t}_{{\rm{end}}},p\right)}\left|\left[{t}_{{\rm{start}}},{t}_{{\rm{end}}}\right)\cap \left[{t}_{i},{t}_{i+1}\right)\right|,$$

where $$\Delta t=| [{t}_{0},{t}_{1})| ={t}_{1}-{t}_{0}$$ is the length of the interval $$[{t}_{0},{t}_{1})$$ and ∩ denotes the intersection of two intervals. Note that most of the terms in the sum (3) are zero, so the feature values can be computed efficiently. In addition to the actual points *p*, a feature is also constructed for the use of the radio. In that case, the sum (3) goes over all the events that use radio communication.

### DMOP Features

DMOP data document events of (anonymized) commands (e.g., 309Q) that are being issued to different (anonymized) subsystems and units (e.g., ATTT). Every DMOP event is given as a triplet $$(t,c,s)$$, where *t* is the start of the command *c*, that was issued to the subsystem/unit *s*. A list of command-groups, grouped by subsystem/unit *s* is provided as a supplementary material. Let $${\mathscr{ {{D}} }}$$ denote the set of all DMOP events. A feature is constructed for every command and its value for the time interval $$[{t}_{i},{t}_{i+1})$$ is

4 $${C}_{c}(i)={\rm{\min }}\left\{{T}_{{\rm{MAX}}},{\rm{\min }}\{{t}_{i}-t\,| \,(t,c{\prime} ,p{\prime} )\in {\mathscr{ {{D}} }}\wedge t\le {t}_{i}\wedge c{\prime} =c\}\right\},$$

where $min\,{\rm{\varnothing }}={\rm{\infty }}$ and ${T}_{{\rm{MAX}}}$ is set to one day. Thus, the value of ${C}_{c}(i)$ is the time since the command *c* has been issued for the last time before the start of the interval *t*_*i*_, with the correction that after *T*_MAX_ time, the value of the feature remains *T*_MAX_.

We construct a similar feature for each subsystem *s*. If ${\mathscr{S}}$ is the set of commands that can be issued to the subsystem, the value of the corresponding feature is

5 $${S}_{s}(i)=\mathop{{\rm{\min }}}\limits_{c\in {\mathscr{S}}}{C}_{c}(i).$$

Lastly, we create binary indicators

6 $${B}_{s}(i)=\left\{\begin{array}{ll}1; & {S}_{s}(i) < {T}_{{\rm{MAX}}}\\ 0; & {S}_{s}(i)\ge {T}_{{\rm{MAX}}}\end{array}\right.,$$

which are interpreted as indicators of whether a given subsystem is active during the time interval $({B}_{i,s}=1)$ or not (${B}_{i,s}=0$).

### EVT and LT Features

Finally, we also construct four additional features. Two are computed from EVT data and give information about the position of MEX in its highly elliptical orbit. Note that the position is given in terms of time, since the raw data are insufficient to apply Kepler’s laws^18^. Thus, for the time interval $$[{t}_{i},{t}_{i+1})$$, the features $${t}_{{\rm{pericenter}}}$$ and $${t}_{{\rm{apocenter}}}$$ give the time until the passing through the next extreme point of the elliptical orbit (either pericenter or apocenter), and the time since the last passing through one of those points. The time differences are computed with respect to the time *t*_*i*_. The feature values are normalized, so that $${t}_{{\rm{pericenter}}}(i)+{t}_{{\rm{apocenter}}}(i)=1$$, i.e., the actual times are divided by the time needed for travelling half of the orbit (see Fig. 1(b)).

The remaining two features are computed from the LT data. These are

- the distance between Sun and Mars,
- the solar constant at Mars.

The values of these features for the time interval $$[{t}_{i},{t}_{i+1})$$ are computed with respect to the time *t*_*i*_ and are obtained by linear interpolation of the values from the raw data. Note that the solar constant is inversely proportional to the square of the $$Sun-Mars$$ distance: To facilitate the use of different ML methods, they are both included in the dataset. One could also resort to using the NASA SPICE system to obtain these values (https://naif.jpl.nasa.gov/naif/).

### Electrical currents

When describing the preprocessing of the values of electrical currents through a given heater, we follow Fig. 1(c). For every time interval $$[{t}_{i},{t}_{i+1})$$, we proceed as follows. First, the measurements that fall within this interval (shown in blue) are identified. Second, the last measurement before the start of the interval (at $${t}_{{\rm{previous}}}\le {t}_{i}$$), and the first measurement after the end of the interval (at $${t}_{{\rm{next}}}\ge {t}_{i+1}$$) are identified (shown in green). Third, the values within the intervals $$({t}_{previous},{t}_{i})$$ and $$({t}_{i+1},{t}_{next})$$ are linearly interpolated including the values at *t*_*i*_ and $${t}_{i+1}$$ (red dots). Let $$EC(t)$$ denote the value of the corresponding curve (shown as a dashed line) at time *t*. The value, identified with the interval $$[{t}_{i},{t}_{i+1})$$, is calculated as the average

7 $$\frac{1}{{t}_{i+1}-{t}_{i}}{\int }_{{t}_{i}}^{{t}_{i+1}}EC(t)\;{\rm{d}}t,$$

i.e., the area under the curve (blue-shaded area), divided by the length of the interval Δ*t*.

The above procedure does not cover rare events where measurements are missing in a given time interval. In such cases, we rely on interpolation of given specific critical-time values ${t}_{{\rm{critical}}}=5\,{\rm{\min }}$, chosen by the spacecraft operators. If the time between the two measurements (marked with green in Fig. 1(c)) is shorter than ${t}_{{\rm{critical}}}$, i.e., ${t}_{next}-{t}_{previous} < {t}_{{\rm{critical}}}$, we perform linear interpolation between these two. Otherwise, if the interval is larger than the critical-time value, the values are marked as ‘missing’ (character‘?’). It is up to the user, whether the corresponding records (row) will be removed from the dataset or further imputed. Similarly, the above procedure is also applied to rare cases where there are no known measurements in a given time interval. Also note that, if no succeeding measurement exists, it is assumed that the value of the current at ${t}_{i+1}$ (the right red value) equals the last known measurement. An analogous procedure is applied in the cases where no preceding measurement exists.

## Data Records

The data consisting of context data and thermal power consumption measurements, capture the status of the spacecraft over the period from 22. 8. 2008 to 14. 4. 2014 (or three Martian years) is sampled at six different time intervals that range from 1 min to 1 hour (60 min). Each data record (i.e., example) in the dataset pertains to a specific time interval, described with features (i.e., telemetry and context data) and target variables (i.e., the electrical current running through the 33 power lines). Table 1 shows the number of data records/examples and the number of features for each time resolution. It also includes the proportion of missing values in the data, which are caused by occasional *MEX–Earth* communication problems that prevent the transmission of (parts of) the data from the spacecraft, and, consequently, prevent the computation of the feature or target values. For evaluation purposes, we suggest using 2/3 of the data for training models and 1/3 of the data for testing (this division of the data corresponds to 2 Martian years vs. 1 Martian year). The data records, for each of the six variants, are available on figshare^19^ in CSV format .

**Table 1:** Summary of the provided datasets at each time resolution: Number of examples, number of features per group, the number of targets, proportion of missing values and dataset size (measured in megabytes (MB)).

resolution (min)	examples	targets	features						proportion of missing values	size [MB]
			dmop	evt	ftl	influx	lt	total		
1	3957119	33	380	2	23	182	2	589	0.094%	17892
5	791424	33	380	2	23	182	2	589	0.094%	3616
10	395712	33	380	2	23	182	2	589	0.095%	1817
15	263808	33	380	2	23	147	2	554	0.100%	1110
30	131904	33	380	2	23	112	2	519	0.110%	503
60	65952	33	380	2	23	77	2	484	0.120%	225

## Technical Validation

MEX, like any other mission, before the actual launch, undergoes several phases of pre-launch test simulations where different parameters of the spacecraft are tested under various conditions. Using these data, various first-principles models are then being developed using both the pre-launch and (subsequently) post-launch data in order to evaluate the behavior of the spacecraft.

With respect to data validation during transmission, once operational the spacecraft uses CRC codes^20^, ensuring data are not changed due to communication errors. The process relies on MUST^21^ – a tool that checks the packets of data for a valid CRC and discards every information with invalid CRCs. Therefore, one can safely assume that the data on the ground (Earth) is the same as the data on-board (MEX). The data is transmitted in frames, that contain packets of raw data which need to be calibrated. The processes of decommutation (unpacking of the packets) and calibration are also handled by MUST. This procedure has been validated with unit tests and more than a decade of operational use by more than 20 missions.

**Fig. 2:**
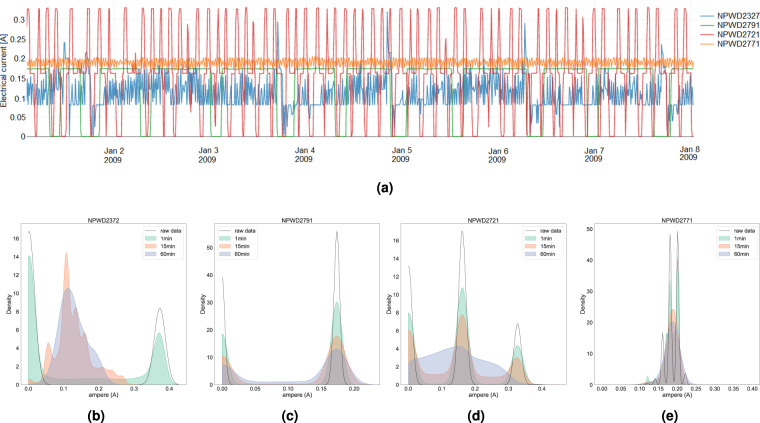
Top: A sample of the real values of the electrical current (in $amperes$) running through four MEX power lines located at different parts of the spacecraft for a selected time window (first week of January 2009), at a 15 min resolution. *Bottom:* Comparison of value distributions (in $amperes$) at different time-resolutions (1 *min*, 15 *min* and 60 *min*) to the unprocessed raw data of four MEX power lines **(b)** NPWD2372, **(c)** NPWD2791, **(d)** NPWD2721, and **(e)** NPWD2771) illustrated in **(a)**. We can see that, in general, the prepossessed data have expected properties: For fast-changing power-lines the modes are joined at higher time-resolutions, whereas for slower it remain similar. From a data-analysis perspective, this further justifies the need of analysing MEX’s behaviour at different time-resolutions.

**Fig. 3:**
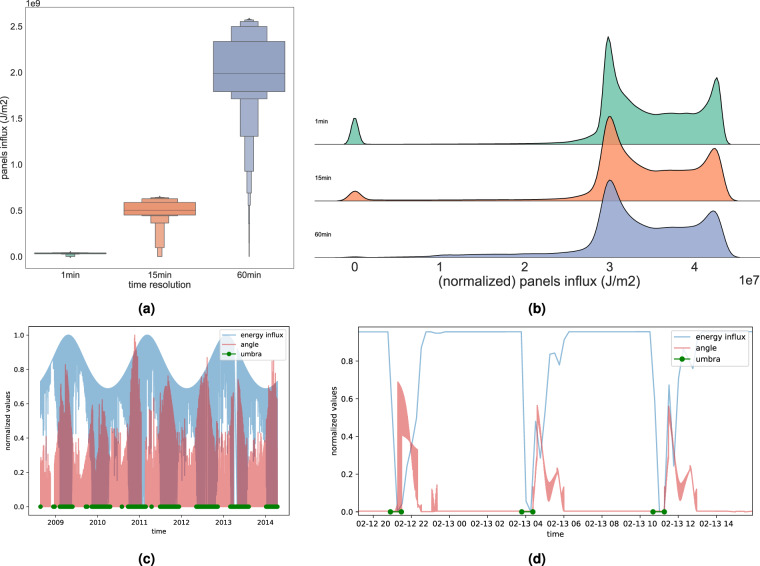
Distributions of a descriptive energy-influx feature panels@influx at different time resolutions (1 *min*, 15 *min* and 60 *min*) in terms of **(a)** box-plots of actual values and **(b)** density plot of normalized values of the influx ($J/{m}^{2}$). panels@influx denotes the influx on the spacecraft’s solar panels. We observe that, as expected, while influx values vary in magnitude between time-resolutions, their distribution properties remain similar. Moreover, **(c)** on a macro-scale (period 2009-2014) the energy-influx measured at the solar panels, panels@influx, depends on the value of the solar constant. However, on a **(d)** micro-scale (12-13th February 2009), the same influx depends more on the angle and occurrence of (pen)umbras. Such behavior is expected for this feature. For visualisation purposes, the values depicted in subfigures (**b**), (**c**) and (**d**) are normalized. Values in (**b**) are normalized to the min-max interval of the 1min dataset, while (**c**) and (**d**) to [0, 1] interval.

Such raw data are the basis of the datasets proposed in this paper. As previously described, this raw data has been cleaned and transformed into a machine-learning-ready format. All six variants of the presented data (per time-resolutions) were inspected and validated by domain experts (engineers operating MEX). Namely, exploratory data analyses of key data properties (such as value ranges, distributions, etc) of the variables, revealed that the transformed data correctly represent the telemetry and power consumption data. Instances of the analyses for the 1 *min*, 15 *min* and 60 *min* resolution datasets are given in Figs. 2 and 3. Namely, Fig. 2 illustrates comparison of value distributions (in $amperes\;(A)$) at different time-resolutions (1 *min*, 15 *min* and 60 *min*) to the unprocessed raw data of four MEX thermal power lines depicted in Fig. 2(a). Figure. 3 presents a comparison of distributions of a descriptive energy-influx feature panels@influx (in $joules\;per\;sq.\;meter\;(J\,/\,{m}^{2})$ at different time resolutions (1 *min*, 15 *min* and 60 *min*). Finally, the data presented in this paper were also inspected for anomalous and outlier values, potentially arising from bad transmissions, and verified against the expected behavior of the spacecraft. All of the tests confirmed the validity of the data at hand.

## Usage Notes

The data at hand are an invaluable resource for safely operating MEX, ensuring its health, and, at the same time, maximizing its scientific return. Thus far, the data have been considered only in the context of predictive modeling: the engineered features were used to predict the electrical currents running through the 33 power lines.

In the first instance, the task of predicting the thermal-power consumption was approached as a task of multi-target regression^14^, with both local and global predictive approaches based on ensembles of predictive clustering trees^22^. The local approaches were used for learning a separate predictive model for each power line, while the global approaches were used for learning a single predictive model for all power lines simultaneously. The same approach was used in the winning solution^14^ of the Kelvins Mars Express Power Challenge (organized by ESA and accessible at https://kelvins.esa.int/mars-express-power-challenge/) on thermal power prediction for MEX^13^, performing substantially better than the typically used handcrafted model.

Next, similar tasks were considered in a more extensive study, that includes a comparison of methods for multi-target regression based on ensembles of predictive clustering trees^22^ and gradient boosted trees^23^. The problem was also approached as a hierarchical multi-target regression task, where the 33 power lines are organized into a hierarchy, which yielded performance improvements^24^.

Furthermore, considering the sheer volume of the data, especially at the resolution of 1 *min*, the problem of the thermal power consumption prediction was formulated as a data stream mining task^25–27^. In this scenario, for obtaining a predictive model, the learning algorithm sees each data example only once. Based on this, the learning algorithm is able to adjust the predictive model and detect potential drifts in data. Note that, in these works, the obtained predictive models were used for short-term forecasting.

While prior work used the data in a narrow predictive modeling setting, there are many potential directions for further exploitation and exploration of these data. First, from a spacecraft-operations point of view, results from analyses on these data are likely to be of interest for designing and initiating analyses on other spacecraft. Second, in a more machine learning context, the data can be used for evaluating approaches for outlier and anomaly detection as well as contextual anomaly detection - these are highly relevant tasks for spacecraft operation. Third, given the temporal nature and volume of the presented data (at different granulates), it can also be used for evaluating data-stream learning methods, especially for change detection and adaptation in time-evolving data streams. Note that real-world datasets of such size and quality, representative for various challenges that might appear in mining data streams, are very rare.

Note that, due to the sensitive and proprietary nature of parts of the data, namely concerning DMOP commands (and units) as well as thermal components, detailed descriptions of some of the variables are not available. While all the other variables are understandable, this can still somewhat limit comprehensible, fully white-box, analyses of the data for users without a particular level of expertise in spacecraft operations. Therefore, for a wider user-base, these data are more suitable for benchmarking ML approaches and pipelines, as well as various aspects of their design. Since the data provided here are in an ML-ready format, it can be readily used with a variety of machine learning toolboxes, such as scikit-learn^28^, CLUS+^29^, WEKA^30^, Orange^31^, KNIME^32^, and MOA^33^. It can be used for further investigation of the thermal power consumption of MEX, to showcase the use of artificial intelligence when optimizing spacecraft operations, or as valuable benchmark datasets for various ML methods from different fields.

## Data Availability

The raw data are available on the ESA website https://kelvins.esa.int/mars-express-power-challenge/ as provided by the MEX operations team at ESOC. These data are pre-processed using the above-described approaches.
